# Editorial: “Humanized” Large Animal Cancer Models: Accelerating Time and Effectiveness of Clinical Trials

**DOI:** 10.3389/fonc.2019.00793

**Published:** 2019-08-27

**Authors:** Kyle M. Schachtschneider, Gregers Jungersen, Lawrence B. Schook, Dhanansayan Shanmuganayagam

**Affiliations:** ^1^Department of Radiology, University of Illinois at Chicago, Chicago, IL, United States; ^2^Department of Health Technology, Technical University of Denmark, Lyngby, Denmark; ^3^Department of Animal Sciences, University of Illinois, Urbana, IL, United States; ^4^Biomedical and Genomic Research Group, Department of Animal Sciences, University of Wisconsin–Madison, Madison, WI, United States

**Keywords:** porcine, canine, cancer, translational, models

In the United States alone, excluding contributions from governmental agencies and academic institutions, the private sector invests between US$1.8–2.6 billion for each drug and between US$75–94 million for each medical device development (1). From inception to regulatory approval, the development process requires ~13 years for drugs and 4.5 years for devices. Despite the time, cost, and effort, new therapies experience an 86–95% failure rate, primarily in the course of human clinical trials (1, 2). Cancer drugs seem to pose the greatest challenge with only 3.4–6.4% of those entering human clinical trials successfully advancing through to clinical use (2).

The potential failure rates, and the associated cost and time, can be mitigated if efficacy and safety of cancer drugs can be validated in translational preclinical animal models that mimic the complexities of the human disease, including comorbidities and confounding factors such as diet. Early discovery of unexpected hurdles allows for redesign and refinement prior to costly clinical testing. Of the three categories of animal models of human disease (3), *predictive models* (effects of a given treatment), *isomorphic models* (similar symptoms, different etiology), and *homologous models* (same symptoms, same etiology), the latter provide the greatest translational value when dealing with complex diseases such as cancers. Similarities to humans in anatomy, physiology, metabolism, immunology, and genetics is essential for recapitulating the interplay between various risk factors and molecular mechanisms of tumor development and progression. Here, an animal that is similar in size to humans adds critical value as it more closely models the size of tumors relative to organ and body size. This is not only important in modeling the spatial physiology of tumor microenvironments and the pathophysiological impact of developing tumors on the whole body, but also for accurately evaluating the efficacy and safety of promising novel therapies. Furthermore, human-sized models allow for the use of human clinical imaging modalities and medical devices during the pre-clinical phase of therapy development, enabling efficacy assessments to be made as they would be in the clinical setting.

As the vast majority of drugs tested in small animal cancer models fail in human clinical trials, there is a need for large animal models to accurately translate results obtained in small animal models to human clinical use, and also address unmet clinical needs. In addition, the majority of preclinical immunotherapy studies conducted in rodents have translated poorly to the clinic due to substantial differences between murine and human immunology. As the porcine and canine immune systems display substantial homology to that of humans, these large animals represent excellent platforms for preclinical investigation of cancer immunotherapies (4). Porcine (5) and canine (6) cancer models are thus rapidly gaining acceptance and popularity for use in cancer research, and are being recognized as valuable tools for testing of drugs and devices in co-clinical trials. The continued development of genomic and phenomic tools and databases also provides the ability through genome editing to create “humanized” experimental large animal models that can support interventional targeted cancer drug and device development. These large animal models also allow for the inclusion of relevant comorbidities such as alcohol-induced cirrhosis, non-alcoholic steatohepatitis (NASH), diabetes, obesity, and cardiovascular disease.

This *Frontiers in Oncology* special issue presents three original research articles, six reviews, and a hypothesis article spanning porcine, canine, and ovine cancer models. In the **Original Research** section, Gray et al. describe a novel naturally-occurring ovine pulmonary adenocarcinoma model to validate the ability of miniaturized implantable sensors to monitor tumor microenvironment by integrating techniques used in the treatment of human lung cancer patients. Schlein et al. use a canine model of naturally-occurring brain cancers, along with human tumor samples and cell lines, to validate procaspase-3 as a druggable target for specific brain tumors, particularly high grade astrocytomas. Boettcher et al. establish that xenotransplantation of human ovarian cancer into severe combined immune deficient (SCID) pigs phenotypically resembled human ovarian carcinomas and substantiate further development of orthotopic pig models.

In the **Mini Review** and **Review** sections, Gray et al. describe the advantages of using naturally occurring ovine pulmonary adenocarcinoma models, including their value in evaluating chemotherapeutic agents and monitoring the tumor microenvironment. Faraji and Gaba review medical imaging modalities, current radiologic diagnostic criteria and response assessment schemes for evaluating therapeutic response and disease progression, and explore translation of radiologic imaging protocols and standards to large animal models of malignant disease. Bailey and Carlson describe a pancreatic tumor model utilizing Cre-inducible transgenic Oncopigs with KRAS and p53-null mutations to overcome the limited translational accuracy and utility of murine models. Duran-Struuck et al. highlight the advantages of swine models for the study of hematological malignancies and describe their experience with a transplantable tumor model that utilizes spontaneously arising tumors in MGH swine. Xu et al. discuss the utility of developing genetically defined porcine cancer models as clinically relevant, personalized cancer models for use in co-clinical trials, ultimately improving treatment stratification and translation of therapeutic approaches to clinical practice. Fan and Selting present the value of dogs with spontaneous tumors as a model to advance harnessing of abscopal effects for clinical use. That is, how radiotherapy could be used to trigger systemic anticancer immune activation, allowing for regression of cancerous lesions distant from the primary site of radiation delivery.

In the **Hypothesis and Theory** section, Boettcher et al. present the hypothesis that SCID pig models are well-suited for improved engraftment and differentiation of human immune cells, and how such humanized pig models can be used to study interactions between human tumors and human immune cells, and to develop patient-specific therapies.

## Author Contributions

All authors listed have made a substantial, direct and intellectual contribution to the work, and approved it for publication.

### Conflict of Interest Statement

The authors declare that the research was conducted in the absence of any commercial or financial relationships that could be construed as a potential conflict of interest.
